# Estimating Contact Rates Between *Metarhizium anisopliae*–Exposed Males With Female *Aedes aegypti*

**DOI:** 10.3389/fcimb.2021.616679

**Published:** 2021-04-29

**Authors:** Filiberto Reyes-Villanueva, Tanya L. Russell, Mario A. Rodríguez-Pérez

**Affiliations:** ^1^ Instituto Politécnico Nacional, Centro de Biotecnología Genómica, Laboratorio de Biomedicina Molecular, Ciudad Reynosa, Mexico; ^2^ Australian Institute of Tropical Health and Medicine, James Cook University, Cairns, QLD, Australia

**Keywords:** males’ releases, *Metarhizium anisopliae*, auto-dissemination, biological control, dengue, *Aedes aegypti*

## Abstract

**Introduction:**

Effective control of *Aedes aegypti* will reduce the frequency and severity of outbreaks of dengue, chikungunya, and Zika; however, control programs are increasingly threatened by the rapid development of insecticide resistance. Thus, there is an urgent need for novel vector control tools, such as auto-dissemination of the entomopathogenic fungi *Metarhizium anisopliae* and *Beauveria bassiana.* The aim of this study was to estimate contact rates of *M. anisopliae*-exposed males with wild female *Ae. aegypti*. As a control the contact rates of untreated males with wild females was contrasted.

**Methods:**

The study was conducted in Reynosa, Mexico. The treatment and control households (n = 15 per group) were geographically separated by an arid and hot area that naturally prevented the flight of males between arms. In each control household, 40 *M. anisopliae*-exposed male *Ae. aegypti* were released per week for 8 weeks (specimens were exposed to a concentration of 5.96 × 10^7^ conidia/cm^2^ for 24 h; n = 4,800 males). In each control household, 40 untreated males were released per week for 8 weeks (n = 4,800 males). All specimens were dust-marked prior to release. Mosquito abundance was monitored with human landing collections, and captured *Ae. aegypti* were examined for any dust-marking.

**Results:**

In the treatment households, the contact rate of *Ae. aegypti* females with marked, fungus-treated males was 14% (n = 29 females marked from 197). Where in the control households, the contact rate of females with marked, untreated males was only 6% (n = 22 marked from 365). In the treatment households the recapture rate of released males was at 5% and higher than that for the control households (which was 2%). Auto-dissemination of *M. anisopliae* from infected males to female *Ae. aegypti* was demonstrated through the recovery of an infected female from the floor of a household.

**Conclusions:**

Overall, the contact rate between *M. anisopliae*-infected males with the natural female population was 60% higher than for the control group of healthy males. The results provide further support to the release of fungus-exposed males as a potentially useful strategy against *Ae. aegypti*, though further research is required.

## Introduction

The global expansion of *Aedes aegypti* and its vectored arboviruses is the widest ever recorded (Kraemer et al., 2015). Vector control is the primary tool used in the fight against arbovirus transmission, but is hampered by inadequate program management, limited human, financial and infrastructural capacity, community apathy to eliminate water-storage containers, plus increased travel and uncontrolled urbanization (Gould et al., 2017). Of serious concern, is the large-scale and intensive use of insecticides for *Aedes* control, which increases the selection pressure on vector populations to develop insecticide resistance. In fact, resistance to all four classes of insecticide commonly used in vector control has been recorded for *Ae. aegypti* (Moyes et al., 2017). In response, novel methods of *Aedes* control are under development and the most promising options are: 1) transinfection with *Wolbachia* (Moreira et al., 2009), 2) incompatible insect treatment (Marris, 2017; Ritchie, 2018), 3) sterile insect treatment (Lees et al., 2015), 4) the use of adult mosquitoes to transfer insecticides (Devine et al., 2009; Mains et al., 2015; Brelsfoard et al., 2019), biological control using *Bacillus thuringiensis* and oomyceto *Leptolegnia chapmanii* (Rodríguez-Pérez et al., 2012), and entomopathogenic fungi, such as *Metarhizium anisopliae* and *Beauveria bassiana* (Scholte et al., 2007; Paula et al., 2008; Reyes-Villanueva et al., 2011; García-Munguía et al., 2011).

*Aedes* control with entomopathogenic fungi has demonstrated promise (Reyes-Villanueva et al., 2011; García-Munguía et al., 2011; Garza-Hernández et al., 2013; Garza-Hernández et al., 2015). Previous laboratory studies have shown that *M. anisopliae* is auto-disseminated from fungus-exposed *Ae. aegypti* males (FEMs) to unexposed females, and can kill 85% of females infected with DENV-2 in less than 10 days (Reyes-Villanueva et al., 2011; Garza-Hernández et al., 2013; Garza-Hernández et al., 2015). In order to gain regulatory acceptance and effectively implement bio-control with the Ma-CBG-2 strain fungus of high virulence at 6 × 10^8^ conidia/mL (LT_50_ of 7.5 ± 0.4 days; which reduced fecundity by up to 99% (Reyes-Villanueva et al., 2011), the lab-to-field development process has an end goal of demonstrating effectiveness and feasibility in the field (Vontas et al., 2014). Although control of *Ae. aegypti* using entomopathogenic fungi has been widely studied in laboratory conditions (*e.g*. stages of infection, formulations, fungal susceptibility against different stages of mosquito development, virulence of different fungal propagules), our method here assessed, *i.e*. the *M. anisopliae *conidia transfer from FEMs to females provided an additional tool for integrated dengue vector control programs through intra-domicile releases of FEMs. Under semi-field conditions, FEMs made over twice mating attempts without insemination than the uninfected males and during both attempts and successful matings, the FEMs were able to transfer the amount of fungus to females, even after the 5th mating (about 10% of male’s conidia load) which was sufficient to kill 50% of females within 3 days (Garza-Hernández et al., 2015). Thus, indicating that there is potential for auto-dissemination of *M. anisopliae* from males to females as a dengue control tool.

The overall aim of this study was to evaluate the contact rates of FEMs to unexposed females in a small-scale field trial. This was measured by releasing either FEMs or unexposed males in the treatment and control arms (*i.e.* a parallel arm experiment), respectively, and examining the ratio of recaptured, dust-marked FEMs and unexposed males to the number of dust-marked females. Thus, we tested the hypothesis whether the contact rates between released males and the natural female population differed between the treatment and control arms.

## Materials and Methods

### Study Site and Period

**Figure 1:**
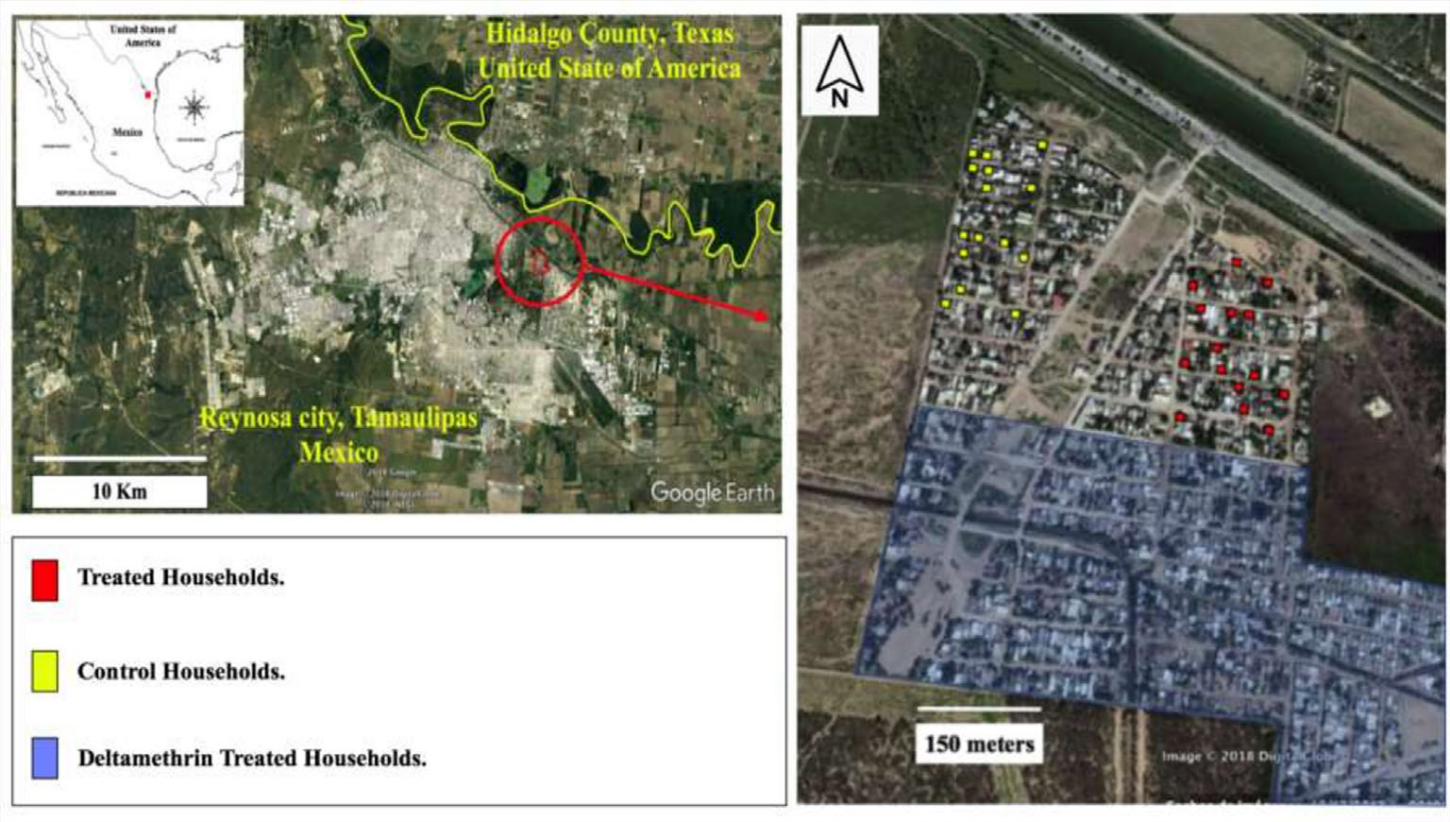
Map including the 30 experimental households at Reynosa, Mexico: Households with releases of *Metarhizium anisopliae -* exposed male *Aedes aegypti* in red-filled circles; Households treated with uninfected males in yellow-filled circles. The 12 blocks with experimental households (with colored circles), and the non-experimental households (in blue) located southward were treated with deltamethrin and cleaned of man-made containers. North, east and west-side of surveyed area are feral/inhabited areas.

The study was conducted in the neighborhood “Nuevo Amanecer” at Reynosa, Mexico (19° 14’ 39.91’’W and 26° 3’ 16.2’’N), located 33 meters above sea level and with 700,000 inhabitants. The climate is hot-dry with an annual mean temperature of 22°C; the dry season lasts around 40 days in July-August with daily temperatures reaching 40°C to 42°C; conversely, the winter encompasses at least 20 days in December to January with minimum temperatures of 0°C to 5°C (INEGI, 2014). The *Ae. aegypti* local seasonality is bimodal with activity in March-June and September-November (Rodriguez-Perez et al., 2020). This study was conducted in October and November of 2016, in a cluster of 120 households arranged in two arms (control and treatment) each separated clearly by an arid and hot field (500 m length, 200 m wide) with only a 10% grass coverage. This field acted as a natural barrier to prevent the dispersal of released males between treated and control arm (**Figure 1**). Preliminary human landing collections were conducted to select 30 households (15 per group) with similar abundances of *Ae. aegypti* to be designated as the experimental households. The experimental households had an average of five female mosquitoes recorded through 2015 (Rodriguez-Perez et al., 2020). The households were distributed across 101,000 m^2^, and were occupied by an average of five residents (**Figure 1**). Southward, the blocks were surrounded by non-experimental households, while eastward and westward, there was a 300-m-wide open grassland field. Northward, there is an 80-m-wide water canal, and beyond there are 4 km of grassland, with *Prosopis* spp. and *Acacia* spp. bushes and few dispersed non-experimental households until the USA boundary wall. Daily temperature and relative humidity in field, measured by waterproof digital thermometer, varied from 18°C to 28°C, from 70% to 85%, respectively, during the survey interval.

### Experimental Design

Ten days before the survey, the low but similar *Ae. aegypti* abundance was verified by human landing collections conducted in the 30 experimental households. Human landing catch was conducted for 20 min per household, and 10 households were covered during 17:00 to 20:30 h. This was repeated across three consecutive days to examine all 30 households. Here, one and zero female *Ae. aegypti* were found in the 15 treated and in the 15 control households, respectively, during the preliminary surveys.

Next, the experimental releases of male *Ae. aegypti* commenced. Each individual release event involved releasing 40 males (either FEMs or unexposed males) per household. Specifically, half (n=20 red-marked males) were released inside the living room of the household, while the other half (n=20 yellow-marked males) were released in the front yard at 3 m from the main entrance of each household. FEMs and unexposed males were marked with the same powder colors (red and yellow). Males were released between 17:00 and 20:30 h and all 30 households were treated in three consecutive days, releasing 200 FEMs in five households and 200 unexposed males in five control households per day per week, during the 8 weeks.

### Insectary Maintenance and Preparation of *Ae. aegypti*

The released males were 4 to 6 days old, unmated, sugar-fed *Ae. aegypti* taken from a colony set up in 2006 with larvae from Monterrey, Mexico and reared following published protocols (Reyes-Villanueva et al., 2011). Briefly, larvae were held at a density of 200 per liter of deionized water in an enamel pan; pupae were confined in a screened cage; females were blood fed on the arm of only one voluntary person.

### Origin and Maintenance of *M. anisopliae* and Production of Conidia

The Ma- CBG-2 strain of *M. anisopliae sensu lato* was isolated from a *Galleria mellonella* exposed, in a plastic cup, to a sample of soil collected at rural habitat around the city of Arteaga, Coahuila, México; then it was cultured on potato-dextrose- agar (PDA) and incubated at 25°C for 20 days to allow sporulation. The Ma-CBG-2 strain was tested at an exposure concentration of *circa* 5.96 × 10^7^ conidia/cm^2^ on a filter paper prepared as previously reported (Reyes-Villanueva et al., 2011). Briefly, the fungus was cultured on potato-dextrose-agar plates incubated at 25 ± 2°C for 20 days in the dark. The conidia yield was estimated by using a mixture of 0.5% Tween-20 and 0.5% Triton-X in 0.85% saline solution. The spore suspension was centrifuged at 3,500 rpm for 10 min and then diluted to 1.6 × 10^8^ conida/mL based on hemocytometer. To facilitate the following experiments, 5 to 7 mL (depending on the conidia harvested using 20 standard Petri dishes) of the final suspension was applied to 8 cm diameter filter papers (2.5 μm pore).

### Exposure of Adult Males to the Fungi

**Figure 2:**
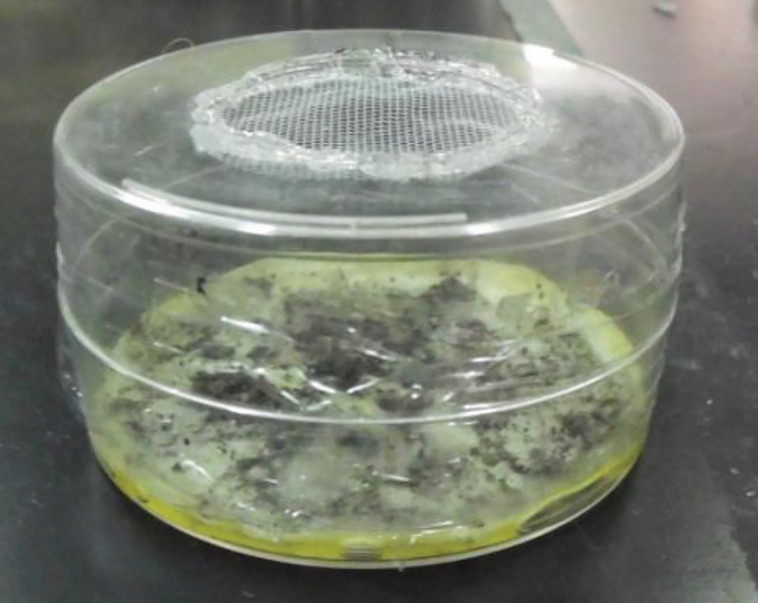
Chamber for exposure of *Aedes aegypti* to *Metarhizium anisopliae* containing a filter at the bottom impregnated with a concentration of 5.96 × 10^7^ conidia cm^2^ of the fungus. Mosquitoes were fed with 5%-sucrose in a cotton ball placed on the hole at top half. Another hole at the lateral dish served for transferring the mosquitoes into the chamber.

Seven mL of the mix of the conidia suspension was poured onto a sterile Whatman filter paper that was placed on the bottom half of a Petri dish and then dried at room temperature (about 24°C) for 24 h. After drying, a second half-dish was placed over the first one to create an exposure chamber as depicted in **Figure 2** (Reyes-Villanueva et al., 2011). Both treated with dry conidia and untreated (clean) filters were placed in chambers (**Figure 2**) also described elsewhere (Garza-Hernández et al., 2015), where 20 males were confined per chamber for 24-h; the confinement was from 11:00 to 11:00 h, then each group of 20 males was transferred to 1-L meshed–cardboard cup where mosquitoes had a “resting” time of 3 h and then were marked with yellow or red dust by the procedure described previously (Garza-Hernández et al., 2015) after 3 h post-treatment. The mosquitoes were kept during and after treatment under insectary conditions which were maintained at 25 ± 1°C, relative humidity of 80 ± 5%, and a photoperiod of 14:10-h L:D. Around the 15:00 h the cups were placed in dry-ice boxes and transported to the field to be released.

### Release of Exposed and Untreated Males, Mosquito Sampling, and Processing

A parallel arm study was used. In the treatment arm, 40 FEMs were released in each experimental household (n = 15) each week; while in the control arm, 40 unexposed males were released weekly in each household (n = 15). The household was the experimental unit and the whole experiment encompassed 8 weeks (October to November). In order to avoid the presence of *Ae. aegypti* larvae in or around the households and to have a similar and low population of mosquitoes at the beginning of the experiment, a month prior to the survey the *Ae. aegypti* population was placed under control pressure. First, in all indoor walls (cement-blocks), closets and bathrooms of each household, deltamethrin (Deltametrina 25, Química del Golfo, Monterrey, México) wettable granules 0.25 mg (with a residual effect of ~ 30 days) was applied at Ultra Low Volume (equipment Cifarelli^®^ model Nuvola 5 horse power, Voghera, Italy) adjusted to 800 mL per minute to delivering an average of 2.4 L (3 min. of 800 mL per min. per household). Then, 3 weeks prior the male releases, backyards of all households were cleaned by removing any water-storage container (this activity took 5 days to complete). The vector control was conducted in each experimental household, as well as all other households of the neighborhood block.

The mosquito abundance in each household was sampled with human landing collections. The human landing collections were conducted in the living rooms of the households and were carried out by a two-person team: one person exposed their upper body, while the other person collected the mosquitoes that had landed on the partner’s exposed skin with a mouth aspirator. Human landing catches were conducted using the same schedule as the mosquito releases, described above, and occurred about 15 min after the mosquitoes were released in each household.

Immediately after each human landing collection, the mosquitoes were examined in the aspirator. When conducting human landing collection for *Ae. aegypti*, it is common to capture both host-seeking females and swarming males (Roth, 1948), thus all individuals were morphologically identified to both species and sex (Darsie and Ward, 2005). Marked mosquitoes were then detected with the aid of an ultra-violet light lamp. The marked males and female mosquitoes captured were counted and released immediately in order not to modify the experimental cohort of *Ae. aegypti* through removal trapping. After completing human landing collection, the floor of each households was examined for the presence of any dead mosquitoes through exhaustive searching. Mosquito cadavers were placed into sterile 5-mL tubes and transported to the lab where they were immersed in 1% chloride for 1 min, dried for 10 min, and then placed into Petri dishes containing PDA for sporulation.

Prior ethical approval was received, see Ethics approval and consent to participate section below.

### Statistical Analysis

A dataset was constructed with numbers of total and marked females, unmarked (wild) males, marked males (FEMs and unexposed males) in red and yellow per household/week/treatment. Marked females and males were compared between treatments by 2 × 2 contingency tables based on the chi-square statistic. The means of females, FEMs, unexposed males and wild males as response variables were compared between treated and control households (arms) by Student’s t *post-hoc* tests conducted with a negative binomial regression model with treatment as class variable with proc glimmix in SAS 9.4 (SAS Institute Inc, 2013).

## Results

### Contact Rate With Females

Overall, there were 197 and 365 wild females collected in treated and control households, respectively (**Table 1**). In the treatment households, the contact rate of *Ae. aegypti* with marked, fungus-exposed males was 14.7% (n = 29). In contrast, the contact rate of females with marked, untreated males was only 6% (n = 22) in the control houses. These percentages represent the proportion of the total females that were dust-marked through auto-dissemination from the released males. Therefore, the capture of marked females was 60% higher in households where FEMs were released (15% = 29/197) than in control households treated with unexposed males (6% = 22/365; χ2 = 11.71, df=1, p =0.0006). Note that the fungus incidence of sampled mosquitoes was not recorded due to specimens being released immediately after capture and identification.

**Table 1:** *Aedes aegypti* documented in human-landing counts conducted in 15 households/week during 8 weeks in treated and control site.

Week	Total females		Marked females^1^		Recaptured males^2,3^		Wild males	
	Control	Treated	Control	Treated	Control	Treated	Control	Treated
1	3	2	1	1	21 (9,12)	36 (17,19)	12	27
2	13	7	2	2	16 (7,9)	33 (17,16)	8	21
3	20	9	2	4	9 (5,4)	42 (19,23)	9	20
4	47	29	6	2	10 (5,5)	26 (14,12)	2	5
5	43	20	4	2	7 (3,4)	24 (11,13)	4	9
6	115	60	1	4	12 (6,6)	41 (17,24)	1	4
7	121	65	5	13	8 (5,3)	19 (10,9)	2	4
8	3	5	1	1	13 (6,7)	22 (12,10)	2	1
Total	365	197	22	29	96 (46,50)	243(117,126)	40	91

^1^The proportion of marked females was 24% higher in treated households where fungus-exposed males were released than in control ones where unexposed marked males were released. (χ2 = 11.71, df =1, p <0.05).

^2^Each column comprises data for 8 weeks and three numbers per week: The number of total recaptured males, then red and yellow males in parenthesis.

^3^The proportion of recaptured males was 60% higher in treated than in control households (χ2 = 66.07, df =1, p < 0.001).

In treated, forty males previously exposed for 24 h to a filter with a dose of 5.2 × 10^6^ conidia per cm^2^ of Metarhizium anisopliae were released per household/week; in control, 40 uninfected males exposed for 24 h to a clean filter, were released per household/week. Total female mosquitoes including marked females, recaptured males containing red and yellow males (in parentheses), and wild males (with no mark), are shown.

### Abundance of Marked Males

A total of 339 dust-marked males (FEMs and unexposed males) were recaptured out of the 9,600 released being an overall recapture rate of 3.5%. In the treated households, the recapture rate was 5% (n = 243/4,800), with a mean/household of 2.02 ± 0.11. In the control households, the recapture rate was significantly less at 2% (96/4,800), with a mean of 0.80 ± 0.22 (t = 4.86, df =378, p<0.001). The males that were released indoors versus outdoors were marked with different color dust. At both treated and control households, relatively equal ratios of males released indoor: outdoor were recaptured, with the ratios being 1:1.07 and 1:1.08 from treated and control households respectively (χ2 = 0.0015, df=1, p =0.96).

### Abundance of Wild Males

A total of 131 unmarked male *Ae. aegypti* were captured. These wild males were 2.2 times more abundant in the treated households with a mean of 0.75 ± 0.11 (n= 91), against a mean of 0.33 ± 0.07 (n = 40) in control households (t = 3.30, df = 238, p <0.001). Note that the abundance of released males far outweighed the wild population, with wild males representing only 27.9% of all captured males (n = 131/470).

### Retrieval of Cadavers

During searches for dead mosquito on the household floors, a total of 17 cadavers were found (16 marked males and one female). Of these, from the red-marked female the fungal surveyed strain was successfully re-isolated; that female was mated.

## Discussion

Conidia of *M. anisopliae* germinate in less than 20 h (Hywel-Jones and Gillespie, 1990) and all FEMs released here, putatively, already had a 27-h infection by direct exposure to the fungus in the chamber (**Figure 2**), which is relevant because an earlier study conducted in a greenhouse reported that FEMs marked with red powder seized the double of female *Ae. aegypti* (7) than the unexposed males marked with yellow powder (3) (Garza-Hernández et al., 2015). Within approximately 4 days post-exposure, 50% of FEMs die (Reyes-Villanueva et al., 2011); so, it seems that once FEMs start to be impacted by fungal infection the response is to prioritize mating over flight for dispersal, which is in line to the predominance of FEMs (60%) in treated households than unexposed males in control households reported here. The increase in mating activity in males challenged by pathogens has been documented in other insects, such as in *Schistocerca gregaria* infected by *M. anisopliae* (Clancy et al., 2017) and in the cricket *Gryllus texensis* threatened by iridovirus (Adamo et al., 2014). During the current experiment it was observed that FEMs, unexposed males, and wild males were all captured in the same swarms formed on a volunteer (bait) during human landing collection. It is known that when some males of *Ae. aegypti* start hovering on a human head, males and females are attracted to the swarm at an intensity contingent on the number of males present in the swarm (Fawaz et al; Roth, 1948); this is linked with the higher capture (2x) of wild males in treated households than in control households.

The effect of fungal infection on the flight of male *Ae. aegypti* remains yet unknown. The low recapture (40%) of marked males in control households is possibly related to higher dispersal of the healthy males after they were released. It is worthy to mention that the ratio of indoor: outdoor marked males was similar for FEMs (1:1.08) and unexposed males (1:1.07), which suggests a similar dispersal of those released indoors or outdoors (Verdonschot and Besse-Lototskaya, 2014). In a prior mark-release-recapture study of untreated male *Ae. aegypti* in Mexico, the maximum recapture rate and distance recorded (by backpack aspirator) were 6.55% (138/2,107) and 166 m (Valerio et al., 2012); nevertheless, they also found that more than 50% of recaptured males were found in the three houses nearest to the release point. In this study, non-experimental households were inter-dispersed between the experimental households, and then possibly the unexposed males migrated to adjacent non-experimental households, which were not surveyed by human landing collections and therefore must be examined in future studies. Last, in a recent study conducted approximately at 15 km (in USA, across the border) from this study site, examined larvae marked with isotopes in tires that produced males collected in BG Sentinel traps at 220 m (Juarez et al., 2010).

## Conclusions

This is the first report about contact rates recorded by mark-release-recapture of *M. anisopliae*-exposed males with female *Ae. aegypti*, in field. Overall, the contact rate between *M. anisopliae*-infected males with the natural female population was 60% higher than for the control group of healthy males. This pilot data provides strong evidence in support of the potential of entomopathogenic fungi to control *Ae. aegypti* through auto-dissemination. The next step in the evaluation process of this tool is to investigate the effectiveness in a large-scale field trial.

## Data Availability Statement

The data set analyzed in the present study is available from the corresponding author upon reasonable request. Requests to access these data sets should be directed to FR-V, frv65@hotmail.es.

## Ethics Statement

The studies involving human participants were reviewed and approved by the Bioethics Committee of the Escuela Nacional de Medicina y Homeopatia of the Instituto Politecnico Nacional (Mexico City) under reference ENMH-CB-061-2013. 202. Written informed consent was obtained from all human volunteers to collect mosquitoes (HLC collectors).

## Author Contributions

FR-V and MR-P conceived and designed the experiment. MR-P conducted field-work. FR-V and TR analyzed the data and wrote the manuscript. All authors contributed to the article and approved the submitted version.

## Funding

This study was funded by Fondo Sectorial SS/IMSS/ISSSTE-CONACyT, grant 200664 (PI MR-P), Mexican Government. MR-P was also supported (publication fees) by Comisión de Operación y Fomento de Actividades Académicas (COFAA) of Instituto Politécnico Nacional (IPN). It was also granted by SIP-IPN (Nos. 20201174 and 20201972).

## Conflict of Interest

The authors declare that the research was conducted in the absence of any commercial or financial relationships that could be construed as a potential conflict of interest.

## Abbreviations

FEMs, fungus-exposed males; L:D, light: dark.
